# Lipid variability and risk of microvascular complications in Action to Control Cardiovascular Risk in Diabetes (ACCORD) trial: A post hoc analysis

**DOI:** 10.1111/1753-0407.13273

**Published:** 2022-06-06

**Authors:** Daniel Nyarko Hukportie, Fu‐Rong Li, Rui Zhou, Jia‐Zhen Zheng, Xiao‐Xiang Wu, Meng‐Chen Zou, Xian‐Bo Wu

**Affiliations:** ^1^ Department of Epidemiology, School of Public Health Southern Medical University Guangzhou China; ^2^ School of Public Health and Emergency Management Southern University of Science and Technology Shenzhen China; ^3^ Department of General Surgery 157^th^ Hospital, General Hospital of Guangzhou Military Command Guangzhou China; ^4^ Department of Endocrinology and Metabolism, Nanfang Hospital Southern Medical University Guangzhou China

**Keywords:** high‐density lipoprotein, low‐density lipoprotein, microvascular complications, remnant cholesterol, triglyceride, 高密度脂蛋白, 低密度脂蛋白, 甘油三酯, 残余胆固醇, 微血管并发症

## Abstract

**Background:**

Greater lipid variability may cause adverse health events among diabetic patients. We aimed to examine the effect of lipid variability on the risk of diabetic microvascular outcomes among type 2 diabetes mellitus patients.

**Methods:**

We assessed the association between visit‐to‐visit variability (measured by variability independent of mean) in high‐density lipoprotein (HDL) cholesterol, low‐density lipoprotein‐cholesterol (LDL), triglyceride, and remnant cholesterol (RC) measurements among participants involved in the Action to Control Cardiovascular Risk in Diabetes (ACCORD) study and the risk of incident microvascular outcomes, including nephropathy, neuropathy, and retinopathy. Cox proportional hazards models were used to estimate the hazard ratios (HRs) and 95% confidence intervals (CIs), adjusted for potential confounders.

**Results:**

There were 2400, 2470, and 2468 cases of nephropathy, neuropathy, and retinopathy during a follow‐up period of 22 600, 21 542, and 26 701 person‐years, respectively. Higher levels of HDL, triglyceride, and RC variability were associated with an increased risk of incident nephropathy and neuropathy. Compared with the lowest quartile, the fully adjusted HRs (95% CI) for the highest quartile of HDL, triglyceride, and RC variability for nephropathy risk were 1.57 (1.22, 2.01), 1.50 (1.18, 1.92), and 1.40 (1.09, 1.80), respectively; and for neuropathy, the corresponding risks were 1.36 (1.05, 1.75), 1.47 (1.14, 1.91), and 1.35 (1.04, 1.74), respectively. Null association was observed between LDL variability and all microvascular complications. Additionally, all associations of variability in the other lipids with retinopathy risk were null.

**Conclusion:**

Among individuals with type 2 diabetes mellitus, HDL, triglyceride, and RC variability were associated with increased risks of nephropathy and neuropathy but not retinopathy. Trial registration: ClinicalTrials.gov., no. NCT00000620.

## Graphical abstract

Highlights

Association of lipid variability with diabetic microvascular complications.

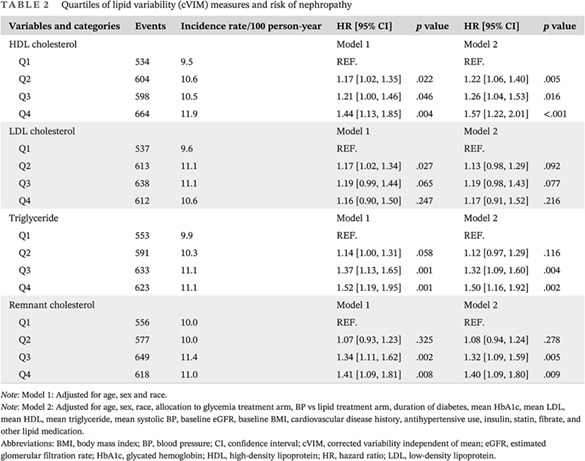

## 摘要

**背景:**

较大的血脂变异性可能会导致糖尿病患者的不良健康事件。我们旨在2型糖尿病(T2DM)患者中研究血脂变异性对糖尿病微血管结果风险的影响。

**方法:**

我们评估了ACCORD研究参与者在高密度脂蛋白胆固醇(HDL), 低密度脂蛋白胆固醇(LDL), 甘油三酯和残余胆固醇(RC)测量中的随访间变异性(通过独立于平均值的变异性来衡量)与发生微血管病变(包括神经病变, 肾病和视网膜病变)的风险之间的关系。使用COX比例风险模型估计危险比(HR)和95%可信区间(CI), 并对潜在混杂因素进行了校正。

**结果:**

在22600人‐年, 21542人‐年和26701人‐年的随访期内, 分别有2400例肾病, 2470例神经病变和2468例视网膜病变。HDL, 甘油三酯和RC变异性越高, 发生肾病和神经病变的风险就越高。与最低四分位数相比, 高密度脂蛋白, 甘油三酯和RC变异性对肾病风险的完全校正后的HR(95%CI)分别为1.57(1.22, 2.01), 1.50(1.18, 1.92)和1.40(1.09, 1.80)；对于神经病变, 相应的风险分别为1.36(1.05, 1.75), 1.47(1.14, 1.91)和1.35(1.04, 1.74)。在LDL变异性和所有微血管并发症之间无明显关联。此外, 其他血脂的变异性与视网膜病变风险之间无明显关联。

**结论:**

在T2DM患者中, HDL, 甘油三酯和RC变异性与肾病和神经病变的风险增加有关, 但与视网膜病变无关。

## 1 INTRODUCTION

Dyslipidemia is a common presentation in individuals with type 2 diabetes mellitus (T2DM), even among those with good glycemic control.^1^ Indeed, individuals with T2DM have been found to have abnormally high levels of triglycerides, decreased high‐density lipoproteins (HDL), and modest or normal levels of low‐density lipoproteins (LDL).^1^ In a cross‐sectional study of T2DM patients, the researchers found the prevalence of hypertriglyceridemia, abnormal HDL levels (<40 mg/dL), and abnormal levels of LDL (>130 mg/dL) to be 61.9%, 54.3%, and 8.9%, respectively,^2^ similar to the findings of other studies.^3^, ^4^ These abnormal lipid levels have been implicated in the development of several diabetes‐related complications including diabetic macrovascular and microvascular disease.^5^, ^6^, ^7^, ^8^

Meanwhile, higher variabilities in lipid variables have also been found to be detrimental to health among populations with or without diabetes.^9^, ^10^, ^11^, ^12^, ^13^, ^14^, ^15^, ^16^ For example, in a study of approximately 1000 participants with clinical presentation of coronary disease involved in the Treating to New Target trial, greater variability in HDL, triglyceride, and LDL was found to be associated with cardiovascular events.^11^ Also, a recent study of over 10 000 T2DM patients showed that higher variability in HDL, LDL, and total cholesterol appeared to increase the risk of all‐cause mortality whereas greater variability in HDL was associated with noncardiovascular deaths.^17^ However, few of these studies were on the relationship between lipid variability and diabetic microvascular complications.^9^, ^14^, ^16^ Diabetic microvascular complications, which comprise nephropathy, neuropathy, and retinopathy, together contribute hugely to the burden of diabetic‐related morbidity and mortality.^18^, ^19^ Reports show that during a lifetime, about 20%‐40% of patients with T2DM would develop diabetic kidney disease,^20^ up to 50% would eventually develop diabetic neuropathy,^21^ and 10% are likely to develop diabetic retinopathy.^22^

With the paucity of studies on the association between lipid variability and microvascular complication, we conducted a post hoc analysis of the Action to Control Cardiovascular Risk in Diabetes (ACCORD) trial to examine the relationship between intraindividual variability in HDL, LDL, triglyceride, and remnant cholesterol (RC) with diabetic microvascular complication. The ACCORD trial was a randomized, multicenter, double 2 × 2 factorial trial in 10 251 participants with T2DM.^23^ The current study gives a unique sample of participants with T2DM, with comprehensive baseline measurements and almost complete follow‐up for diabetic microvascular complications, including nephropathy, neuropathy, and retinopathy.

## 2 METHODS

### 2.1 Study design and subjects

ACCORD was a randomized clinical trial of 10 251 participants with type 2 diabetes who were followed to assess the health effects of intensive glycemic, lipid, and blood pressure (BP) control as against standard control.^24^, ^25^ The design and main results of the ACCORD study have been published previously.^25^ In brief, the ACCORD trial had three study arms: (1) glycemia trial (glycated hemoglobin [HbA1c] <6.0% vs 7.0% < HbA1c <7.9%), (2) lipid trial (fenofibrate vs placebo), and (3) BP trial (systolic BP <120 mm Hg vs systolic BP <140 mm Hg), with all participants involved in the glycemia trial.^26^ Recruitment of participants into the study began in January 2001 through to October 2005 from 77 clinical sites across North America (ie, the United States and Canada). Participants in the ACCORD study were followed up until June 2009.^27^ Ethical approval for the ACCORD study was granted by institutional review boards of each clinical site and written informed consent was obtained from all recruited participants (trial registration: ClinicalTrials.gov., no. NCT00000620).^28^

**FIGURE 1:**
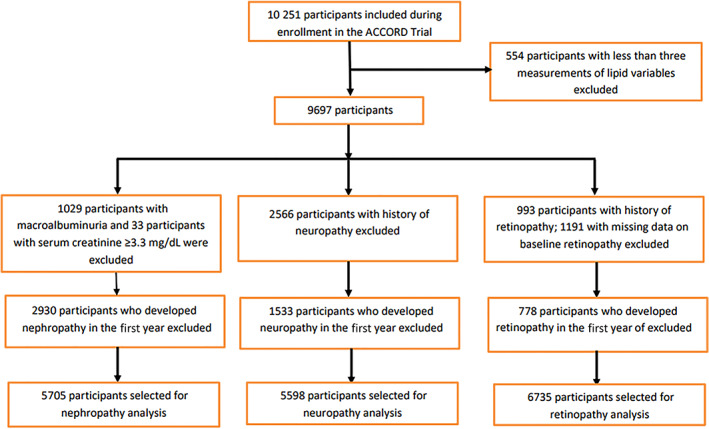
Inclusion and exclusion criteria of participants for lipid variability analysis. ACCORD, Action to Control Cardiovascular Risk in Diabetes

This current study was a post hoc analysis of data available for participants who did or did not participate in the lipid trial arm of the ACCORD study. In order to demonstrate the impact of lipid variability on microvascular complications in T2DM patients, participants with less than three lipid measurements (*n* = 554) were excluded. Then, for each microvascular outcome, individuals with the prevalent microvascular outcome at baseline and those who developed the outcome within the first year of the study were excluded from the analysis of the specific microvascular outcome; that is, for nephropathy outcome, the exclusion would be 1029 participants with prevalent macroalbuminuria, 33 with serum creatinine >3.3 mg/dL, and 2930 who developed incident nephropathy in the first year of the study (total exclusion = 3992); for neuropathy outcome, the exclusion would be 2566 participants with prevalent neuropathy and 1533 participants who developed incident neuropathy in the first year of the study (total exclusion = 4099); and for retinopathy, the exclusion would be 993 patients with retinopathy, 1191 patients with missing record on baseline retinopathy variable, and 778 patients who developed incident retinopathy within the first year of the study (total exclusion = 2962). The analytic samples for nephropathy, neuropathy, and retinopathy outcomes were 5705, 5598, and 6735, respectively (Figure 1).

### 2.2 Definition of lipid variability

During the ACCORD trial, a fasting plasma lipid profile was measured at baseline, then at 4 months, 8 months, 12 months, and yearly thereafter. We calculated remnant cholesterol as total cholesterol ‐ LDL ‐ HDL.^15^ Lipid variability was defined as the variation in HDL, LDL, triglyceride, and RC values between visits. Three indices of variability were used: (1) SD, (2) coefficient of variation (CV), and (3) variability independent of the mean (VIM). VIM was calculated as 100 × SD/mean^β^, where β is the regression coefficient, based on the natural logarithm of the SD on the natural logarithm of the mean. The uncorrected VIM was corrected using this formula: (VIM uncorrected × mean of CV) ÷ mean of VIM uncorrected. Because SD and CV are correlated with the mean level, the corrected VIM (cVIM) was used as the primary variability measure in this study^12^ and CV and SD were used as a sensitivity analysis.

### 2.3 Study outcomes

The endpoints considered in our analysis were microvascular events defined as neuropathy (a composite of (1) new score of >2.0 on the Michigan Neuropathy Screening Instrument, (2) new loss of vibratory sensation [tested using 128 Hz tuning fork], (3) new loss of ankle jerk during Jendrassik maneuver, or (4) new loss of light touch [as measured by 10 g force monofilament test]); nephropathy (a composite of (1) development of macroalbuminuria, (2) development of renal failure, or (3) doubling of baseline serum creatinine or more than 20 mL/min/1.73 m^2^ decrease in estimated glomerular filtration rate [eGFR]), and retinopathy (a composite of (1) retinal photocoagulation or vitrectomy to treat retinopathy, (2) eye surgery for cataract extraction, (3) three‐line change in visual acuity [as measured using Log MAR visual acuity chart], or (4) severe vision loss [as measured by Snellen fraction <20/200]). Patients who experienced any one of the predefined microvascular events that comprised the composite neuropathy, nephropathy, and retinopathy outcome were considered to have experienced that specific disease. Table S1 shows the three predefined ACCORD microvascular end points and their frequency of assessment.

### 2.4 Statistical analysis

Participants' characteristics were described using mean ± SD (SD) or median (25% and 75%) for continuous variables, depending on whether the data distribution was normal (assessed by the Shapiro‐Wilk test); categorical variables were described by frequency and percentage. The association between HDL, LDL, triglyceride, and RC variability (as measured by cVIM) and the risk of outcomes was evaluated by using the quartiles of HDL, LDL, triglyceride, and RC variability.

Cox proportional hazards regression model was used to estimate the hazard ratios (HRs) and 95% confidence intervals (CIs), using HDL, LDL, triglyceride, and RC as a time‐dependent covariate. Two models were used for each outcome. The basic model was adjusted for demographic factors and the full model was adjusted for demographic variables, lipid profile variables (mean values of HDL, LDL and triglyceride), and lipid medication use plus other covariates with *p* values <.05 in the univariate analysis (Table 1). Specifically, model 1 was adjusted for baseline age (continuous), sex (male or female), and race (white or non‐white). Model 2 for nephropathy was adjusted for model 1 plus glycemia treatment arm (intensive or standard), BP vs lipid arm (standard BP, intensive BP, fibrate, and placebo), duration of diabetes, mean HbA1c, mean HDL, mean LDL, mean triglyceride, mean systolic BP, baseline eGFR, baseline body mass index (BMI), cardiovascular disease (CVD) history (yes or no), insulin use (yes or no), antihypertensives (yes or no), statin (yes or no), fibrate (yes or no), and other lipid medications (yes or no). For neuropathy, model 2 was adjusted for model 1 plus glycemia allocation, duration of diabetes, mean HDL, mean LDL, mean triglyceride, baseline BMI, cigarette smoking (yes or no), insulin, statin, fibrate, and other lipid medications. Model 2 for retinopathy was adjusted for model 1 plus glycemia allocation, BP vs lipid arm, duration of diabetes, mean values of HDL, LDL, triglyceride, and systolic BP, baseline BMI, insulin, statin, fibrate, and other lipid medications.

**TABLE 1:** Differences in baseline characteristics of participants with nephropathy, neuropathy, and retinopathy

Characteristics	Nephropathy			Neuropathy			Retinopathy		
	No cases (n = 3305)	Cases (n = 2400)	*p* value*	No cases (n = 3128)	Cases (n = 2470)	*p* value*	No cases (n = 4267)	Cases (n = 2468)	*p* value*
Age, y	62.2 (57.6, 67.4)	62.3 (58.1, 67.4)	.31	61.6 (57.4, 66.3)	62 (57.6, 67.5)	<.001	60.9 (57, 65.6)	62.8 (58.3, 67.8)	<.001
Sex			<.001			.051			.062
Male	1994 (60.3)	1556 (64.8)		1856 (59.3)	1529 (61.9)		2708 (63.5)	1510 (61.2)	
Race			.079			<.001			.023
White	2058 (62.3)	1549 (64.5)		1632 (52.2)	1620 (65.6)		2715 (63.6)	1638 (66.4)	
Glycemia arm			.003			.006			.020
Standard	1628 (49.3)	1277 (53.2)		1495 (47.8)	1271 (51.5)		2072 (48.6)	1271 (51.5)	
Intensive	1677 (50.7)	1123 (46.8)		1633 (52.2)	1199 (48.5)		2195 (51.4)	1197 (48.5)	
Arm of trial			<.001			.16			.001
Standard BP	981 (29.7)	503 (21.0)		689 (22.0)	597 (24.2)		907 (21.3)	618 (25.0)	
Intensive BP	693 (21.0)	563 (23.5)		751 (24.0)	558 (22.6)		971 (22.8)	576 (23.3)	
Lipid placebo	1132 (34.3)	766 (31.9)		828 (26.5)	670 (27.1)		1205 (28.2)	641 (26.0)	
Lipid fibrate	499 (15.1)	568 (23.7)		860 (27.5)	645 (26.1)		1184 (27.7)	633 (25.6)	
Duration of diabetes, y	9 (5, 14)	10 (5, 15)	<.001	8 (4, 14)	8 (5, 14)	.037	8 (4, 13)	9 (5, 15)	<.001
Hba1c, %	8 (7.5, 8.7)	8.1 (7.6, 8.9)	<.001	8.0 (7.5, 8.8)	8.1 (7.6, 8.8)	.23	8 (7.5, 8.7)	8 (7.5, 8.8)	.23
TC, mg/dL	178 (154.5, 206)	178 (154, 204)	.47	179 (153, 208)	178 (155, 207)	.57	177 (153, 206)	178 (154, 206)	.63
HDL, mg/dL	41 (35, 49)	39 (34, 47)	<.001	41 (34, 49)	40 (34, 47)	.011	39 (34, 47)	40 (34, 48)	<.001
LDL, mg/dL	102 (82, 125)	100 (81, 123)	.11	101 (82, 127)	101 (81, 124)	.42	99 (80, 123)	99 (81, 124.5)	.28
Triglyceride, mg/dL	150 (104, 215.5)	160 (111, 230)	<.001	150 (102, 224)	157 (108, 224)	.032	161 (111, 238)	157 (109, 229.5)	.017
RC, mg/dL	30 (21, 43)	32 (22, 46)	<.001	30 (20, 45)	31 (22, 45)	.031	32 (22, 47)	31 (22, 46)	.017
SBP, mm Hg	133 (123, 144)	135 (125, 146)	<.001	136 (125, 147)	135 (125, 146)	.28	134 (124, 145)	135 (124, 146)	.027
BMI, kg/m^2^	31.6 (28.2, 35.8)	32.1 (28.4, 35.9)	.014	31.0 (27.5, 35.0)	31.7 (28.1, 35.6)	<.001	32.1 (28.4, 36.2)	31.7 (28.1, 35.7)	.016
eGFR, mL/min/1.73 m^2^	80.3 (68.8, 92.1)	89 (75.3, 103)	<.001	‐	‐	‐	‐	‐	‐
CVD history			.002			.92			.47
Yes	1031 (31.2)	841 (35.0)		1017 (32.5)	806 (32.6)		1433 (33.6)	850 (34.4)	
Cigarette smoking			.78			.025			.24
Yes	428 (13.0)	317 (13.2)		440 (14.1)	297 (12.0)		640 (15.0)	344 (13.9)	
Insulin use			.004			.018			<.001
Yes	1081 (32.7)	872 (36.3)		841 (26.9)	735 (29.8)		1205 (28.2)	832 (33.7)	
Antihypertensives			.015			.31			.85
Yes	2776 (84.0)	2072 (86.3)		2655 (84.9)	2072 (83.9)		3632 (85.1)	2105 (85.3)	
Statin			.70			.54			.77
Yes	2092 (63.5)	1531 (64.0)		1987 (63.8)	1552 (63.0)		2774 (65.3)	1611 (65.6)	
Fibrate			<.001			.67			.95
Yes	260 (7.9)	129 (5.4)		166 (5.3)	138 (5.6)		301 (7.1)	173 (7.0)	
Other lipid medications			.46			.013			.065
Yes	80 (2.4)	51 (2.1)		52 (1.7)	65 (2.6)		85 (2.0)	34 (1.4)	

*Note*: Data are shown as medians (interquartile range) for continuous variables and as frequencies and percentages for categorical variables.

Abbreviations: BMI, body mass index; CVD, cardiovascular disease; eGFR, estimated glomerular filtration rate; HbA1c, glycated hemoglobin; HDL, high density lipoprotein; LDL, low density lipoprotein; RC, remnant cholesterol; SBP, systolic blood pressure; TC, total cholesterol.

* *p* values for continuous variables are from the rank‐sum test. All other *p* values are from the Pearson chi‐square test.

Other measures of variability, such as CV and SD, were used for sensitivity analyses; we also excluded participants who developed serious adverse events during the first 18 months of the study to test the robustness of the results. Furthermore, the effect of Hba1c and systolic BP variability on the study outcomes was examined by adjusting for Hba1c and systolic BP variability in all the full models. All statistical analyses were two sided, and we considered a *p* value of <.05 to be statistically significant. All analyses were performed using Stata (version 14.2; StataCorp, College Station, Texas).

## 3 RESULTS

Table 1 shows the distribution of participants' characteristics according to whether they developed nephropathy, neuropathy, and retinopathy or not. Compared with participants who did not develop nephropathy, those who developed nephropathy were more likely to be men, belonged to the standard glycemia treatment arm, have a longer duration of diabetes, higher levels of BMI, HbA1c, SBP, triglyceride, RC, eGFR, but had lower levels of HDL, use antihypertensives, were less likely to have CVD, and were less likely to use fibrate and insulin. Participants who developed neuropathy were more likely to be older, of the white race, belonged to the standard glycemia treatment arm, had higher levels of triglyceride, RC, BMI, but had lower levels of HDL, and were less likely to use insulin. In addition, participants who developed retinopathy were more likely to be older, belonged to the white race, assigned to the standard treatment arm, had a longer duration of diabetes, higher levels of HDL, SBP, but lower levels of BMI, triglyceride, RC, eGFR, and were less likely to be on insulin. (Table 1). ACCORD recorded 2400, 2470, and 2468 cases of nephropathy, neuropathy, and retinopathy for a follow‐up period of 22 600, 21 542, and 26 701 person‐years, respectively (Table S2).

The rates of any nephropathy event generally increased with increasing quartiles of mean HDL, LDL, triglyceride, and RC as measured by cVIM (Figure S1). Also, the rates of neuropathy events generally increased with increasing quartiles of mean HDL, triglyceride, and RC (Figure S2) but for retinopathy outcome, an increase in rates was observed only for increasing quartiles of triglyceride and RC variability (Figure S3).

After controlling for potential confounding factors, higher levels of HDL, triglyceride, and RC variability measured by cVIM were associated with increased nephropathy risk. The HR and 95% CI for the highest quartile of HDL, triglyceride, and RC variability in the fully adjusted models were 1.57 (95% CI 1.22, 2.01), 1.50 (95% CI 1.16, 1.92), and 1.40 (95% CI 1.09, 1.80) for nephropathy, respectively compared with each lowest quartile (Table 2). However, the association of the highest quartile of LDL variability with incident nephropathy was not statistically significant (Table 2). When other variability measures were used as the supplementary analysis, higher variability in HDL, triglyceride, and RC measured by CV and SD was associated with an increased risk of nephropathy (Table S3). Also, the association of LDL variability as measured by CV and SD with incident nephropathy remained statistically insignificant (Table S3). The results also remained unchanged after excluding participants who developed serious adverse effects in the first 18 month of the study (Table S4).

**TABLE 2:** Quartiles of lipid variability (cVIM) measures and risk of nephropathy

Variables and categories	Events	Incidence rate/100 person‐year	HR [95% CI]	*p* value	HR [95% CI]	*p* value
HDL cholesterol			Model 1		Model 2	
Q1	534	9.5	REF.		REF.	
Q2	604	10.6	1.17 [1.02, 1.35]	.022	1.22 [1.06, 1.40]	.005
Q3	598	10.5	1.21 [1.00, 1.46]	.046	1.26 [1.04, 1.53]	.016
Q4	664	11.9	1.44 [1.13, 1.85]	.004	1.57 [1.22, 2.01]	<.001
LDL cholesterol			Model 1		Model 2	
Q1	537	9.6	REF.		REF.	
Q2	613	11.1	1.17 [1.02, 1.34]	.027	1.13 [0.98, 1.29]	.092
Q3	638	11.1	1.19 [0.99, 1.44]	.065	1.19 [0.98, 1.43]	.077
Q4	612	10.6	1.16 [0.90, 1.50]	.247	1.17 [0.91, 1.52]	.216
Triglyceride			Model 1		Model 2	
Q1	553	9.9	REF.		REF.	
Q2	591	10.3	1.14 [1.00, 1.31]	.058	1.12 [0.97, 1.29]	.116
Q3	633	11.1	1.37 [1.13, 1.65]	.001	1.32 [1.09, 1.60]	.004
Q4	623	11.1	1.52 [1.19, 1.95]	.001	1.50 [1.16, 1.92]	.002
Remnant cholesterol			Model 1		Model 2	
Q1	556	10.0	REF.		REF.	
Q2	577	10.0	1.07 [0.93, 1.23]	.325	1.08 [0.94, 1.24]	.278
Q3	649	11.4	1.34 [1.11, 1.62]	.002	1.32 [1.09, 1.59]	.005
Q4	618	11.0	1.41 [1.09, 1.81]	.008	1.40 [1.09, 1.80]	.009

*Note*: Model 1: Adjusted for age, sex and race.

*Note*: Model 2: Adjusted for age, sex, race, allocation to glycemia treatment arm, BP vs lipid treatment arm, duration of diabetes, mean HbA1c, mean LDL, mean HDL, mean triglyceride, mean systolic BP, baseline eGFR, baseline BMI, cardiovascular disease history, antihypertensive use, insulin, statin, fibrate, and other lipid medication.

Abbreviations: BMI, body mass index; BP, blood pressure; CI, confidence interval; cVIM, corrected variability independent of mean; eGFR, estimated glomerular filtration rate; HbA1c, glycated hemoglobin; HDL, high‐density lipoprotein; HR, hazard ratio; LDL, low‐density lipoprotein.

Similarly, higher variability in HDL, triglycerides, and RC was positively associated with an increased risk of neuropathy in the full models. Compared with the lowest quartile, the fully adjusted HRs (95% CIs) for the highest quartile of HDL, triglyceride, and RC variability were 1.36 (95% CI 1.05, 1.75), 1.47 (95% CI 1.14, 1.91), and 1.35 (95% CI 1.04, 1.74) for any neuropathy event (Table 3). However, the association between the highest quartiles of LDL variability and neuropathy was consistently null (Table 3).

**TABLE 3:** Quartiles of lipid variability (cVIM) measures and risk of neuropathy

Variables and categories	Events	Incidence rate/100 person‐year	HR [95% CI]	*p* value	HR [95% CI]	*p* value
HDL cholesterol			Model 1		Model 2	
Q1	572	10.8	REF.		REF.	
Q2	625	11.6	1.16 [1.01, 1.32]	.036	1.18 [1.03, 1.36]	.017
Q3	634	11.7	1.23 [1.02, 1.49]	.030	1.26 [1.04, 1.52]	.018
Q4	639	11.7	1.32 [1.02, 1.70]	.032	1.36 [1.05, 1.75]	.020
LDL cholesterol			Model 1		Model 2	
Q1	590	11.1	REF.		REF.	
Q2	632	11.9	1.14 [1.00, 1.31]	.053	1.13 [0.99, 1.30]	.079
Q3	632	11.6	1.19 [0.99, 1.44]	.069	1.16 [0.96, 1.41]	.122
Q4	616	11.2	1.22 [0.94, 1.57]	.132	1.16 [0.90, 1.51]	.253
Triglyceride			Model 1		Model 2	
Q1	559	10.3	REF.		REF.	
Q2	649	12.4	1.33 [1.16, 1.53]	<.001	1.34 [1.16, 1.53]	<.001
Q3	638	11.7	1.34 [1.11, 1.63]	.003	1.36 [1.12, 1.65]	.002
Q4	624	11.5	1.46 [1.13, 1.89]	.004	1.47 [1.14, 1.91]	.003
Remnant cholesterol			Model 1		Model 2	
Q1	551	10.2	REF.		REF.	
Q2	663	12.7	1.32 [1.15, 1.51]	<.001	1.31 [1.14, 1.50]	<.001
Q3	620	11.3	1.21 [1.00, 1.47]	.050	1.23 [1.01, 1.49]	.039
Q4	636	11.7	1.33 [1.02, 1.72]	.032	1.35 [1.04, 1.74]	.024

*Note*: Model 1: Adjusted for age, sex and race.

*Note*: Model 2: Adjusted for age, sex, race, allocation to glycemia treatment arm, duration of diabetes, mean LDL, mean HDL, mean triglyceride, baseline BMI, cigarette smoking, insulin, statin, fibrate, and other lipid medications.

Abbreviations: BMI, body mass index; CI, confidence interval; cVIM, corrected variability independent of mean; HDL, high‐density lipoprotein; HR, hazard ratio; LDL, low‐density lipoprotein.

A similar trend was also observed when CV was used in the supplementary analysis. CV of HDL, triglyceride, and RC was associated with an increased risk of neuropathy, but when SD was used, only the SD of triglyceride and RC was associated with an increased risk of neuropathy (Table S5). The results remained the same as the main analysis using cVIM after excluding participants who developed serious adverse effects in the first 18 months of the study (Table S6).

For retinopathy, higher variability in triglyceride and RC were significantly associated with an increased risk of retinopathy in the basic model (adjusted for age, sex, and race). However, these statistically significant associations were lost in the fully adjusted models (Table 4). A similar trend was confirmed in the sensitivity analysis when CV or SD was used as the variability measure (Table S7). Results remained materially the same after excluding participants with any serious adverse event that occurred in the first 18 months of follow‐up (Table S8).

**TABLE 4:** Quartiles of lipid variability (cVIM) measures and risk of retinopathy

Variables & categories	Events	Incidence rate/100 person‐year	HR [95% CI]	*p* value	HR [95% CI]	*p* value
HDL cholesterol			Model 1		Model 2	
Q1	667	9.1	REF.		REF.	
Q2	697	9.3	1.09 [0.95, 1.25]	.207	1.11 [0.97, 1.27]	.129
Q3	713	9.4	1.12 [0.93, 1.35]	.237	1.14 [0.95, 1.38]	.171
Q4	736	9.7	1.22 [0.96, 1.56]	.110	1.23 [0.96, 1.58]	.099
LDL cholesterol			Model 1		Model 2	
Q1	677	9.1	REF.		REF.	
Q2	710	9.6	1.04 [0.91, 1.19]	.546	1.05 [0.92, 1.21]	.450
Q3	695	9.0	1.00 [0.83, 1.20]	.981	1.02 [0.85, 1.23]	.833
Q4	731	9.5	1.00 [0.78, 1.28]	.978	1.02 [0.79, 1.30]	.887
Triglyceride			Model 1		Model 2	
Q1	659	8.9	REF.		REF.	
Q2	707	9.4	1.07 [0.94, 1.23]	.300	1.07 [0.93, 1.22]	.356
Q3	745	9.9	1.18 [0.98, 1.42]	.076	1.16 [0.96, 1.40]	.113
Q4	702	9.2	1.18 [0.92, 1.51]	.203	1.18 [0.92, 1.52]	.199
Remnant cholesterol			Model 1		Model 2	
Q1	650	8.8	REF.		REF.	
Q2	706	9.4	1.07 [0.93, 1.22]	.348	1.07 [0.93, 1.23]	.323
Q3	745	9.8	1.22 [1.01, 1.47]	.037	1.20 [0.99, 1.44]	.059
Q4	712	9.4	1.20 [0.93, 1.54]	.158	1.21 [0.94, 1.56]	.137

*Note*: Model 1: Adjusted for age, sex, and race.

*Note*: Model 2: Adjusted for age, sex, race, allocation to glycemia treatment arm, BP vs lipid treatment arm, duration of diabetes, mean LDL, mean HDL, mean triglyceride, mean systolic BP, baseline BMI, insulin, statin, fibrate and other lipid medications.

Abbreviations: BMI, body mass index; CI, confidence interval; cVIM, corrected variability independent of mean; HDL, high‐density lipoprotein; HR, hazard ratio; LDL, low‐density lipoprotein.

Also, the results remained materially the same for all the study outcomes after further adjusting for variability in HbA1c and systolic BP in the full models (Table S9).

## 4 DISCUSSION

In this post hoc study of individuals with T2DM who took part in the ACCORD trial, we found higher levels of HDL, triglyceride, and RC variability to be associated with an increased risk of nephropathy and neuropathy, whereas a nonsignificant association was observed between all lipid variability measures and retinopathy after adjusting for potential confounding factors. This study provides epidemiologic evidence regarding the associations between visit‐to‐visit variabilities in lipid measures and the risk of diabetic nephropathy and neuropathy, which can be useful in delineating microvascular risk in individuals with T2DM.

Greater visit‐to‐visit fluctuation in lipid profiles has been reported to negatively affect renal function in individuals with or without T2DM.^16^, ^29^ In a study of 457 individuals with T2DM who were followed up for about 6.8 years, the researchers found triglyceride variability (defined by SD) to be significantly associated with incident microalbuminuria.^9^ Also, Chang et al found HDL variability to be associated with an increased risk of nephropathy progression in a sample of 864 T2DM patients who were followed up for a mean of 3.8 years,^16^ similar to the findings assessing the association of lipid variability with a decrease in GFR among over 7000 diabetic patients.^30^ However, these studies were on a relatively small sample size^9^, ^16^ or limited by lack of a centralized laboratory assessment.^30^ Using data from a large sample of participants with T2DM (*n* = 6735) in a randomized, multicenter, double 2 × 2 factorial trial, we confirmed that HDL and triglyceride variability may be associated with renal function decline.^9^, ^16^, ^29^, ^30^ Our study also found higher variability of RC to be associated with nephropathy risk. Studies on RC variability and risk of nephropathy are limited. A study of patients with type 1 diabetes mellitus (T1DM) did not find an association between RC variability and progression of renal function decline in their full model, although a statistically significant relationship was found between RC concentration and nephropathy progression.^15^ Differences in lipid profiles between T1DM and T2DM may account for the conflicting results of RC variability and nephropathy in our study and others. For instance, whereas patients with T2DM may have decreased HDL, T1DM patients are more likely to have normal or even high levels of HDL unless under inadequate glycemic control.^31^

Contrary to the results of some studies reporting a positive link between LDL variability and renal function decline among individuals with T2DM,^14^, ^30^ our study did not find LDL variability to be associated with incident nephropathy. The reason for this unexpected null association is unknown, but we could speculate that because some participants received lipid treatment with the aim of maintaining good control of LDL levels, the potentially harmful effect of high variability in LDL may have been mitigated. Of note is that a study using a large sample of Korean participants with T2DM (*n* = 13 800) and nondiabetic controls (*n* = 185 898) observed a nonsignificant association between higher variability of LDL (measured by average real variability) and CVD.^32^ Similarly, in a recent 5‐year follow‐up study of 4475 nondiabetic Chinese participants, the researchers found a nonsignificant association between LDL variability and incident T2DM.^33^ Further studies may be necessary to clarify the discordant results of the relationship of LDL variability with adverse health outcomes, including renal function decline. Overall, our study adds to the evidence that high HDL, triglyceride, and RC variability may be associated with an increased risk of nephropathy.

The exact mechanism underlining the pathological link between increased variability in HDL, triglyceride, and RC and some specific microvascular is not clear. However, it has been suggested that abnormal lipid levels may directly affect renal function by triggering harmful renal lipid disturbance and indirectly through a general inflammatory process or oxidative stress, vascular injuries, and alterations in signaling molecules for normal renal function.^34^ For example, hypertriglyceridemia, which is a key manifestation of dyslipidemia among patients with T2DM, has been shown to trigger the secretion of inflammatory mediators and have a proapoptotic effect on the endothelium of the kidney in vitro and vivo.^9^, ^35^

Our results showed a significant association between greater HDL, triglyceride, and RC variability and neuropathy risk, whereas LDL variability did not. There seems to be a lack of data on the relationship between lipid profile variability and peripheral neuropathy; however, some studies have found a significant association between glycemic variability and neuropathy.^36^, ^37^ That notwithstanding, dyslipidemia has been identified to be a risk factor for peripheral and autonomic neuropathy.^38^, ^39^, ^40^, ^41^ In a 13‐year follow‐up longitudinal study of 1533 diabetic patients, the authors found lower levels of HDL and LDL to be significantly associated with a higher risk of polyneuropathy.^42^ Because abnormal levels of lipid profile may be indicative of instability in lipid control (dyslipidemia), a similar deleterious effect may be influenced by increased lipid variability on sensory cells in individuals with diabetes. Although the pathophysiological mechanism underlying the link between dyslipidemia and neuropathy is not yet clear, studies on animal models suggest that impaired lipid metabolism in the presence of dyslipidemia may impair normal mitochondrial processes in dorsal root ganglia and affect mitochondrial size of neurons.^43^, ^44^, ^45^ Furthermore, demyelination as a result of dyslipidemia has also been suggested as another plausible mechanism for lipid‐induced injury of neurons, which may contribute to the development of neuropathy.^46^

Meanwhile, our analysis for the relationship between all variability measures of lipid profile with retinopathy was null. Not many studies have explored the relationship between lipid variability and retinopathy among individuals with T2DM to corroborate our results. However, our results agree with one study that also found variability of RC not to be related to retinopathy among 5150 T1DM patients.^15^ The reason for the null association between all lipid profile variability and retinopathy is not clear, but it could be due to the duration required for lipid variability to cause recognizable pathological changes in the eye. On the whole, the incidence of retinopathy was lower at the close of the study compared to the incidence of nephropathy and neuropathy, suggesting that the rate of developing retinopathy was slower among our study population. Hence, potential risk factors such as dyslipidemia and for that matter, lipid variability linked to the pathogenesis of nephropathy and neuropathy may probably take a longer duration to cause recognizable pathological changes in the retina. Further epidemiological studies may be required to verify these results.

The strength of our study includes the use of a relatively large number of subjects from the ACCORD trial, which had a formalized follow‐up lipid measurement schedule. The use of three variability measures adds to the robustness of the study. There was also a special examination of microvascular complications at baseline and subsequent visits, which permitted the accurate assessment of the effect of lipid variability on microvascular complication risk. However, some limitations also need to be considered: (1) the lipid treatment patients received during the trial may affect lipid variability—however, to address this limitation, we additionally adjusted for various lipid treatments in our full model; (2) because participants in the ACCORD study may have more advanced diabetes with associated comorbidities, an extension of findings to healthier and younger T2DM patients should be done cautiously; and (3) the observational nature of this study can only imply an association but not causation, other residual or unrecorded confounding factors may have contributed to the observed results.

In conclusion, long‐term greater variability in HDL, triglyceride, and RC were associated with the risk of incident nephropathy and neuropathy but not retinopathy among individuals with T2DM. Healthcare providers may target lower variability in lipid (HDL, triglyceride, and RC) in patients with T2DM in order to reduce the risk of microvascular complications.

## AUTHOR CONTRIBUTION

Daniel Nyarko Hukportie and Fu‐Rong Li conceived the study, analyzed the data, and wrote the manuscript. Rui Zhou, Jia‐Zhen Zheng, Xiao‐Xiang Wu, Meng‐Chen Zou, Xand ian‐Bo Wu contributed to the discussion and reviewed/edited the manuscript. All authors contributed to the article and approved the submitted version.

## FUNDING INFORMATION

This study was supported by the National Natural Science Foundation of China (82173607), the Guangdong Basic and Applied Basic Research Foundation (2021A1515011684), Open Project of the Guangdong Provincial Key Laboratory of Tropical Disease Research (2020B1212060042) and Guangzhou Science and Technology Project (202102080597). Funders had no role in the design of the study, the analysis and interpretation of data, and the writing of the paper.

## CONFLICT OF INTEREST

The authors declare that the research was conducted in the absence of any commercial or financial relationships that could be construed as a potential conflict of interest.

## ETHICS APPROVAL AND CONSENT TO PARTICIPATE

The study was reviewed and approved by ACCORD Protocol Review Committee appointed by National Heart, Lung, and Blood Institute (NHLBI) and local institutional review boards of participating clinical sites. The patients/participants provided their written informed consent to participate in this study.

## Supporting information


**Appendix S1** Supporting Information

## Data Availability

The data sets generated and/or analyzed during the current study are available in the Biologic Specimen and Data Repository, https://biolincc.nhlbi.nih.gov/studies/accord/
