# Supplementary material for: Lipid variability and risk of microvascular complications in Action to Control Cardiovascular Risk in Diabetes (ACCORD) trial: A post hoc analysis
Source: J Diabetes. 2022 Jun 6;14(6):365–76. doi: 10.1111/1753-0407.13273 (PMC9366577; doi:10.1111/1753-0407.13273)
Supplement: Supplementary file 1 — Appendix S1 Supporting Information [file JDB-14-365-s001.docx]

**Supplementary Material**

**Supplementary Table 1** Definition of Microvascular outcomes and their frequency of assessment

| Outcome category | Outcome | Definition | Assessment frequency |
| --- | --- | --- | --- |
| Nephropathy | 1 | Development of macro-albuminuria (urinary albumin creatinine ratio>=300mg/g) | Every 4 month |
|  | 2 | Doubling of baseline serum creatinine or more than 20mL/min/1.73 m^2^ decrease in estimated glomerular filtration rate | Annually |
|  | 3 | Renal failure OR end stage renal disease (dialysis) OR serum creatinine>3.3 mg/dL in absence of an acute reversible cause | Every 4 month |
| Neuropathy | 1 | New score of >2.0 on the Michigan Neuropathy Screening Instrument (MNSI) | Annually |
|  | 2 | New loss of vibratory sensation (tested using 128 Hz tuning fork) | Annually |
|  | 3 | New loss of ankle jerk during Jendrassik maneuver. | Annually |
|  | 4 | New loss of light touch (as measured by 10 gm force monofilament test) | Annually |
| Retinopathy | 1 | Retinal photocoagulation or vitrectomy to treat retinopathy | Annually |
|  | 2 | Eye surgery for cataract extraction. | Annually |
|  | 3 | Three‐line change in visual acuity (as measured using Log MAR visual acuity chart). | Biannually |
|  | 4 | Severe vision loss (as measured by Snellen fraction <20/200). | Biannually |

**Supplementary Table** **2** Follow-up time for microvascular outcomes

| Outcome | Total observation | Cases | Total follow-up time in person-years |
| --- | --- | --- | --- |
| Nephropathy | 5705 | 2400 | 22599.8 |
| Neuropathy | 5598 | 2470 | 21542.1 |
| Retinopathy | 6735 | 2468 | 26700.5 |


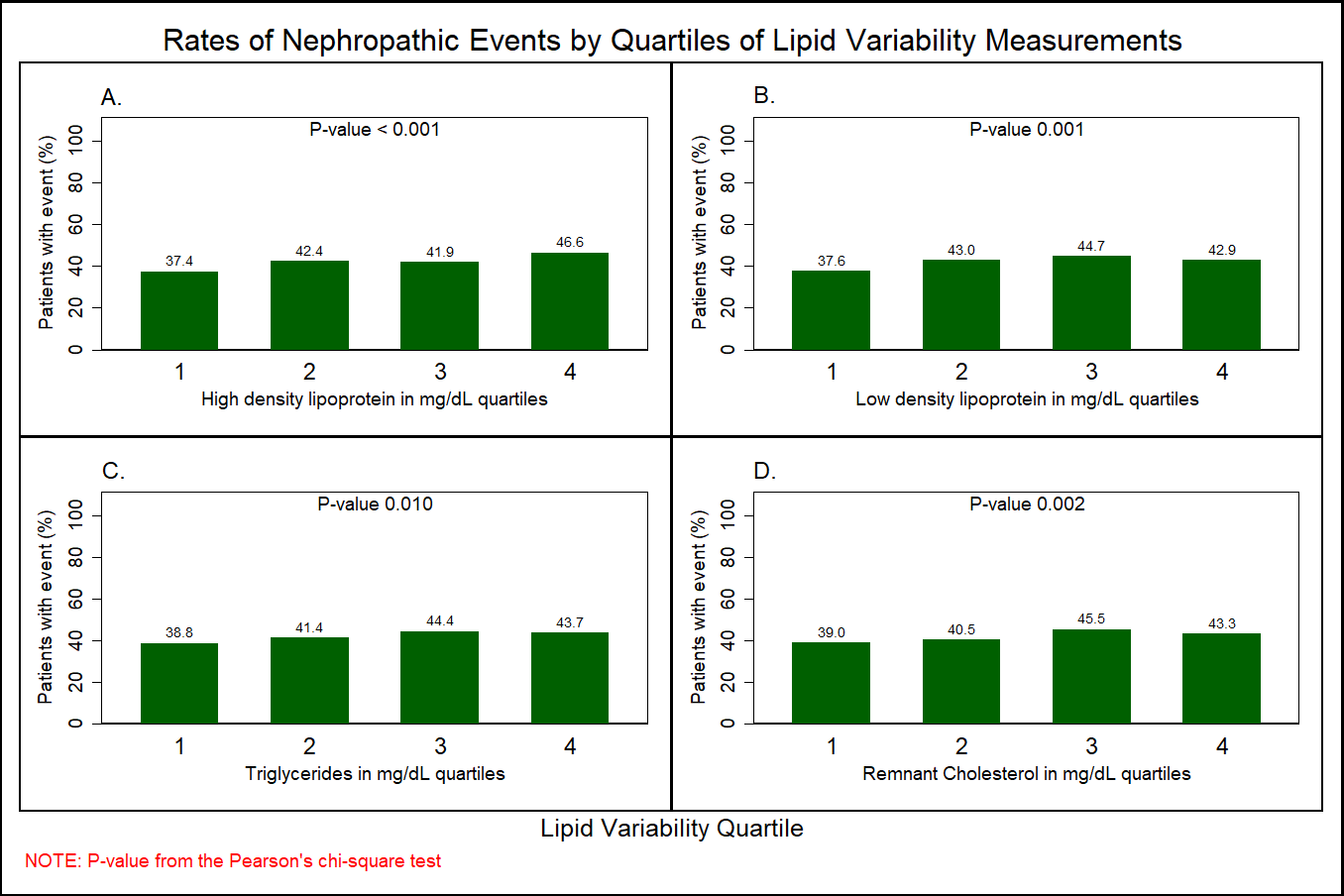


**Supplementary Figure 1** Rates of any nephropathic event in quartiles of high-density lipoprotein (A), low density lipoprotein (B), triglyceride (C) and remnant cholesterol (D) as measured by corrected variability independent of mean (cVIM).


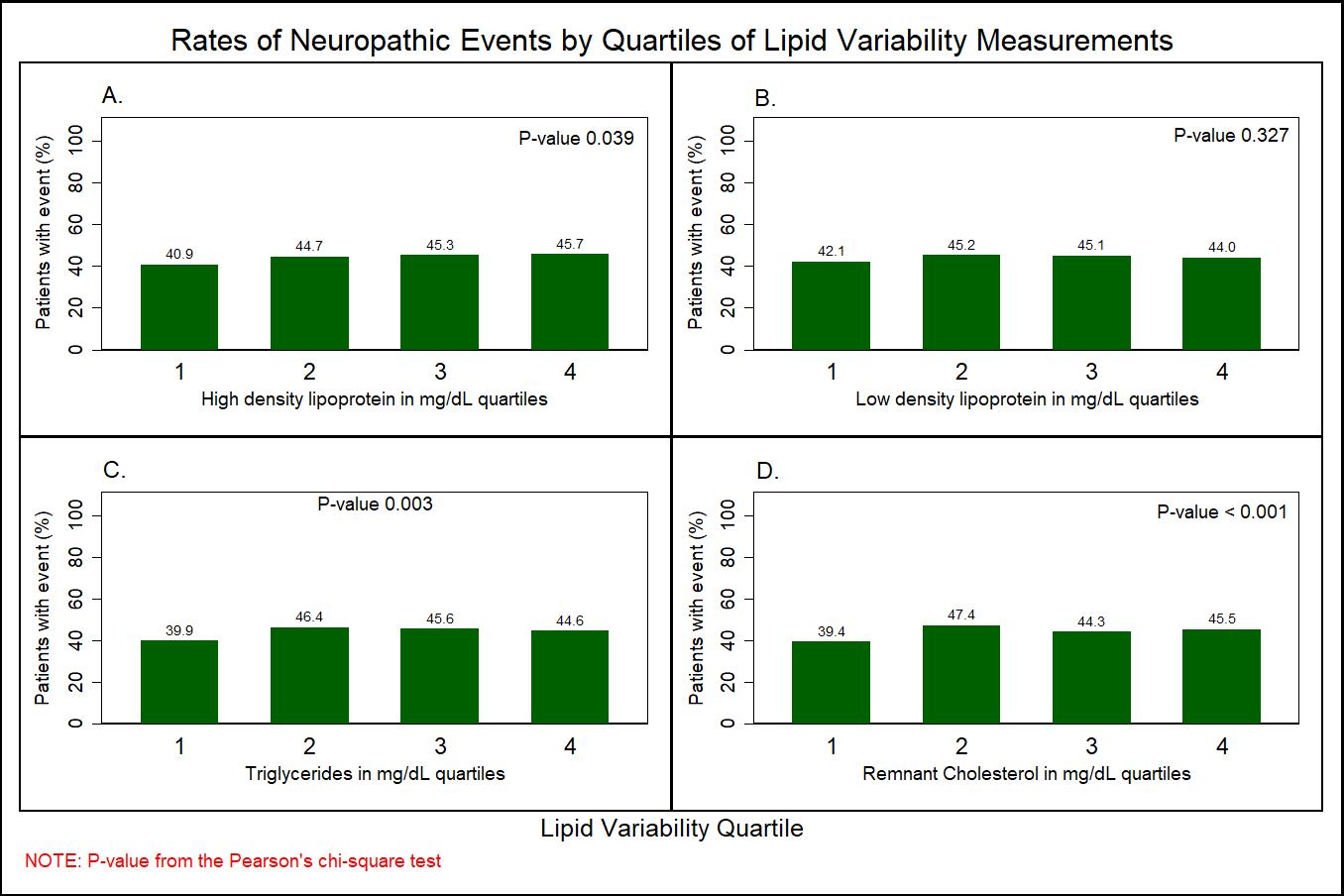


**Supplementary Figure 2** Rates of any nephropathic event in quartiles of high density lipoprotein (A), low density lipoprotein (B), triglyceride (C) and remnant cholesterol (D) as measured by corrected variability independent of mean (cVIM).


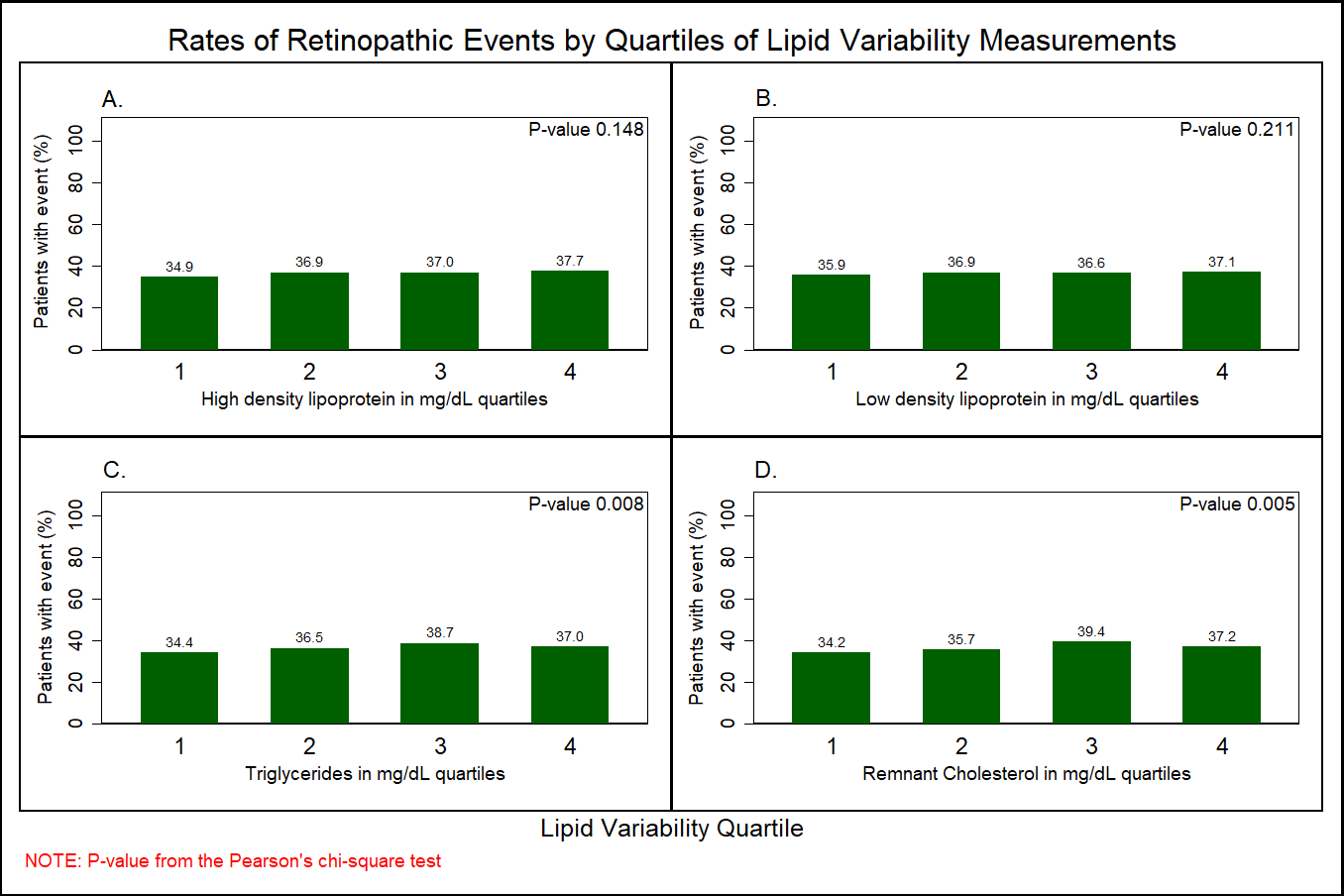


**Supplementary Figure 3** Rates of any nephropathic event in quartiles of high-density lipoprotein (A), low density lipoprotein (B), triglyceride (C) and remnant cholesterol (D) as measured by corrected variability independent of mean (cVIM).

**Supplementary Table 3** Quartiles of lipid variability measures and risk of nephropathy

| Variables & categories | Events | Incidence rate / 100 person-yr | HR [95% CI] | P-value |  | HR [95% CI] | P-value |
| --- | --- | --- | --- | --- | --- | --- | --- |
| HDL Cholesterol |  |  | Model 1 |  |  | Model 2 |  |
| **CV** |  |  |  |  |  |  |  |
| Q1 | 529 | 9.4 | REF. |  |  | REF. |  |
| Q2 | 581 | 10.2 | 1.11 [0.97, 1.27] | 0.142 |  | 1.11 [0.97, 1.28] | 0.130 |
| Q3 | 618 | 10.8 | 1.22 [1.01, 1.47] | 0.037 |  | 1.25 [1.03, 1.51] | 0.022 |
| Q4 | 672 | 12.1 | 1.42 [1.11, 1.82] | 0.006 |  | 1.45 [1.13, 1.87] | 0.003 |
|  |  |  |  |  |  |  |  |
| **SD** |  |  |  |  |  |  |  |
| Q1 | 580 | 10.6 | REF. |  |  | REF. |  |
| Q2 | 608 | 10.7 | 1.05 [0.91, 1.20] | 0.511 |  | 1.10 [0.96, 1.26] | 0.192 |
| Q3 | 603 | 10.6 | 1.08 [0.89, 1.30] | 0.433 |  | 1.17 [0.97, 1.41] | 0.101 |
| Q4 | 609 | 10.6 | 1.12 [0.87, 1.44] | 0.371 |  | 1.35 [1.05, 1.75] | 0.020 |
|  |  |  |  |  |  |  |  |
| LDL Cholesterol |  |  | Model 1 |  |  | Model 2 |  |
| **CV** |  |  |  |  |  |  |  |
| Q1 | 527 | 9.4 | REF. |  |  | REF. |  |
| Q2 | 613 | 11.0 | 1.20 [1.04, 1.37] | 0.011 |  | 1.15 [1.00, 1.32] | 0.056 |
| Q3 | 655 | 11.6 | 1.28 [1.06, 1.55] | 0.010 |  | 1.27 [1.05, 1.54] | 0.014 |
| Q4 | 605 | 10.5 | 1.20 [0.93, 1.55] | 0.164 |  | 1.20 [0.92, 1.55] | 0.173 |
|  |  |  |  |  |  |  |  |
| **SD** |  |  |  |  |  |  |  |
| Q1 | 544 | 9.9 | REF. |  |  | REF. |  |
| Q2 | 625 | 11.2 | 1.12 [0.98, 1.29] | 0.090 |  | 1.07 [0.93, 1.23] | 0.324 |
| Q3 | 616 | 10.7 | 1.07 [0.88, 1.29] | 0.501 |  | 1.05 [0.87, 1.27] | 0.604 |
| Q4 | 615 | 10.6 | 1.05 [0.82, 1.35] | 0.700 |  | 1.10 [0.85, 1.43] | 0.479 |
|  |  |  |  |  |  |  |  |
| Triglyceride |  |  | Model 1 |  |  | Model 2 |  |
| **CV** |  |  |  |  |  |  |  |
| Q1 | 522 | 9.3 | REF. |  |  | REF. |  |
| Q2 | 598 | 10.4 | 1.19 [1.04, 1.37] | 0.014 |  | 1.15 [1.00, 1.32] | 0.053 |
| Q3 | 643 | 11.4 | 1.40 [1.16, 1.69] | <0.001 |  | 1.34 [1.10, 1.62] | 0.003 |
| Q4 | 637 | 11.4 | 1.50 [1.17, 1.93] | 0.002 |  | 1.40 [1.08, 1.81] | 0.010 |
|  |  |  |  |  |  |  |  |
| **SD** |  |  |  |  |  |  |  |
| Q1 | 531 | 9.3 | REF. |  |  | REF. |  |
| Q2 | 575 | 10.0 | 1.10 [0.96, 1.26] | 0.178 |  | 1.08 [0.94, 1.24] | 0.303 |
| Q3 | 614 | 10.8 | 1.25 [1.03, 1.51] | 0.022 |  | 1.27 [1.05, 1.55] | 0.015 |
| Q4 | 680 | 12.5 | 1.52 [1.18, 1.95] | 0.001 |  | 1.48 [1.13, 1.94] | 0.004 |
|  |  |  |  |  |  |  |  |
| Remnant Cholesterol |  |  | Model 1 |  |  | Model 2 |  |
| **CV** |  |  |  |  |  |  |  |
| Q1 | 539 | 9.6 | REF. |  |  | REF. |  |
| Q2 | 585 | 10.2 | 1.13 [0.98, 1.29] | 0.095 |  | 1.10 [0.96, 1.27] | 0.181 |
| Q3 | 651 | 11.5 | 1.38 [1.14, 1.67] | 0.001 |  | 1.34 [1.11, 1.62] | 0.003 |
| Q4 | 625 | 11.2 | 1.45 [1.12, 1.87] | 0.004 |  | 1.38 [1.07, 1.78] | 0.012 |
|  |  |  |  |  |  |  |  |
| **SD** |  |  |  |  |  |  |  |
| Q1 | 528 | 9.3 | REF. |  |  | REF. |  |
| Q2 | 576 | 10.0 | 1.10 [0.96, 1.27] | 0.165 |  | 1.08 [0.93, 1.24] | 0.310 |
| Q3 | 626 | 11.1 | 1.27 [1.05, 1.54] | 0.012 |  | 1.28 [1.06, 1.56] | 0.012 |
| Q4 | 670 | 12.2 | 1.45 [1.13, 1.87] | 0.003 |  | 1.41 [1.08, 1.84] | 0.012 |
| Abbreviations: CV: Coefficient of variation. SD: Standard deviation HR: Hazard ratio. CI: confidence interval.  Model 1: Adjusted for age, sex, race  Model 2: Adjusted for age, sex, race, allocation to glycemia treatment arm, BP vs lipid treatment arm, duration of diabetes, mean HbA1c, mean LDL, mean HDL, mean triglyceride, mean systolic BP, baseline eGFR, baseline BMI, cardiovascular disease history, antihypertensive use, insulin, statin, fibrate and other lipid medications | | | | | | | |

**Supplementary Table** **4** Quartiles of lipid variability (cVIM) measures and risk of nephropathy after excluding participants who developed serious adverse events within the first 18month of follow-up

| Variables & categories | Events | Incidence rate / 100 person-yr | HR [95% CI] | P-value |  | HR [95% CI] | P-value |
| --- | --- | --- | --- | --- | --- | --- | --- |
| HDL Cholesterol |  |  | Model 1 |  |  | Model 2 |  |
| Q1 | 531 | 9.5 | REF. |  |  | REF. |  |
| Q2 | 600 | 10.7 | 1.17 [1.02, 1.35] | 0.024 |  | 1.22 [1.06, 1.40] | 0.005 |
| Q3 | 595 | 10.5 | 1.20 [1.00, 1.45] | 0.053 |  | 1.26 [1.04, 1.52] | 0.018 |
| Q4 | 657 | 11.8 | 1.42 [1.11, 1.82] | 0.006 |  | 1.54 [1.20, 1.98] | 0.001 |
|  |  |  |  |  |  |  |  |
| LDL Cholesterol |  |  | Model 1 |  |  | Model 2 |  |
| Q1 | 532 | 9.6 | REF. |  |  | REF. |  |
| Q2 | 610 | 11.1 | 1.17 [1.02, 1.34] | 0.025 |  | 1.13 [0.98, 1.30] | 0.090 |
| Q3 | 635 | 11.1 | 1.19 [0.98, 1.44] | 0.072 |  | 1.18 [0.98, 1.43] | 0.088 |
| Q4 | 606 | 10.6 | 1.15 [0.88, 1.48] | 0.293 |  | 1.16 [0.90, 1.50] | 0.256 |
|  |  |  |  |  |  |  |  |
| Triglyceride |  |  | Model 1 |  |  | Model 2 |  |
| Q1 | 551 | 9.9 | REF. |  |  | REF. |  |
| Q2 | 588 | 10.3 | 1.14 [0.99, 1.31] | 0.059 |  | 1.12 [0.97, 1.28] | 0.121 |
| Q3 | 629 | 11.1 | 1.36 [1.13, 1.65] | 0.001 |  | 1.32 [1.09, 1.60] | 0.004 |
| Q4 | 615 | 11.0 | 1.50 [1.17, 1.93] | 0.001 |  | 1.48 [1.15, 1.90] | 0.002 |
|  |  |  |  |  |  |  |  |
| Remnant Cholesterol |  |  | Model 1 |  |  | Model 2 |  |
| Q1 | 554 | 10.0 | REF. |  |  | REF. |  |
| Q2 | 575 | 10.0 | 1.08 [0.94, 1.24] | 0.303 |  | 1.09 [0.94, 1.24] | 0.250 |
| Q3 | 644 | 11.4 | 1.34 [1.11, 1.61] | 0.003 |  | 1.31 [1.09, 1.59] | 0.005 |
| Q4 | 610 | 11.0 | 1.40 [1.09, 1.80] | 0.009 |  | 1.39 [1.08, 1.80] | 0.010 |
| Abbreviations: cVIM: Corrected variability independent of mean. HR: Hazard ratio. CI: confidence interval.  Model 1: Adjusted for age, sex and race  Model 2: Adjusted for age, sex, race, allocation to glycemia treatment arm, BP vs lipid treatment arm, duration of diabetes, mean HbA1c, mean LDL, mean HDL, mean triglyceride, mean systolic BP, baseline eGFR, baseline BMI, cardiovascular disease history, antihypertensive use, insulin, statin, fibrate and other lipid medications | | | | | | | |

**Supplementary Table 5** Quartiles of lipid variability measures and risk of neuropathy

| Variables & categories | Events | Incidence rate / 100 person-yr | HR [95% CI] | P-value |  | HR [95% CI] | P-value |
| --- | --- | --- | --- | --- | --- | --- | --- |
| HDL Cholesterol |  |  | Model 1 |  |  | Model 2 |  |
| **CV** |  |  |  |  |  |  |  |
| Q1 | 557 | 10.2 | REF. |  |  | REF. |  |
| Q2 | 633 | 11.5 | 1.16 [1.01, 1.32] | 0.036 |  | 1.19 [1.04, 1.37] | 0.013 |
| Q3 | 641 | 11.6 | 1.24 [1.03, 1.50] | 0.025 |  | 1.25 [1.04, 1.52] | 0.020 |
| Q4 | 648 | 11.6 | 1.34 [1.04, 1.73] | 0.024 |  | 1.36 [1.05, 1.76] | 0.018 |
|  |  |  |  |  |  |  |  |
| **SD** |  |  |  |  |  |  |  |
| Q1 | 565 | 10.5 | REF. |  |  | REF. |  |
| Q2 | 630 | 11.4 | 1.09 [0.95, 1.25] | 0.217 |  | 1.11 [0.97, 1.27] | 0.139 |
| Q3 | 647 | 11.7 | 1.16 [0.96, 1.40] | 0.127 |  | 1.19 [0.99, 1.44] | 0.071 |
| Q4 | 637 | 11.4 | 1.20 [0.93, 1.54] | 0.167 |  | 1.26 [0.97, 1.63] | 0.080 |
|  |  |  |  |  |  |  |  |
| LDL Cholesterol |  |  | Model 1 |  |  | Model 2 |  |
| **CV** |  |  |  |  |  |  |  |
| Q1 | 567 | 10.4 | REF. |  |  | REF. |  |
| Q2 | 641 | 11.8 | 1.10 [0.96, 1.25] | 0.187 |  | 1.08 [0.94, 1.24] | 0.282 |
| Q3 | 640 | 11.5 | 1.14 [0.95, 1.38] | 0.161 |  | 1.12 [0.92, 1.35] | 0.259 |
| Q4 | 631 | 11.3 | 1.18 [0.92, 1.52] | 0.199 |  | 1.11 [0.86, 1.44] | 0.414 |
|  |  |  |  |  |  |  |  |
| **SD** |  |  |  |  |  |  |  |
| Q1 | 577 | 10.7 | REF. |  |  | REF. |  |
| Q2 | 674 | 12.6 | 1.20 [1.05, 1.37] | 0.008 |  | 1.18 [1.04, 1.36] | 0.012 |
| Q3 | 627 | 11.3 | 1.14 [0.94, 1.38] | 0.171 |  | 1.14 [0.94, 1.38] | 0.197 |
| Q4 | 601 | 10.5 | 1.11 [0.85, 1.43] | 0.447 |  | 1.10 [0.84, 1.43] | 0.488 |
|  |  |  |  |  |  |  |  |
| Triglyceride |  |  | Model 1 |  |  | Model 2 |  |
| **CV** |  |  |  |  |  |  |  |
| Q1 | 533 | 9.6 | REF. |  |  | REF. |  |
| Q2 | 644 | 11.8 | 1.23 [1.08, 1.42] | 0.003 |  | 1.24 [1.08, 1.42] | 0.002 |
| Q3 | 648 | 11.7 | 1.28 [1.06, 1.56] | 0.011 |  | 1.30 [1.07, 1.58] | 0.008 |
| Q4 | 654 | 11.9 | 1.38 [1.07, 1.78] | 0.014 |  | 1.41 [1.09, 1.83] | 0.010 |
|  |  |  |  |  |  |  |  |
| **SD** |  |  |  |  |  |  |  |
| Q1 | 543 | 9.7 | REF. |  |  | REF. |  |
| Q2 | 634 | 11.5 | 1.15 [1.00, 1.32] | 0.044 |  | 1.17 [1.01, 1.34] | 0.032 |
| Q3 | 654 | 11.9 | 1.27 [1.05, 1.54] | 0.014 |  | 1.33 [1.09, 1.62] | 0.005 |
| Q4 | 648 | 11.9 | 1.32 [1.02, 1.71] | 0.032 |  | 1.44 [1.09, 1.89] | 0.011 |
|  |  |  |  |  |  |  |  |
| Remnant Cholesterol |  |  | Model 1 |  |  | Model 2 |  |
| **CV** |  |  |  |  |  |  |  |
| Q1 | 538 | 9.7 | REF. |  |  | REF. |  |
| Q2 | 646 | 11.9 | 1.25 [1.09, 1.43] | 0.002 |  | 1.25 [1.09, 1.44] | 0.001 |
| Q3 | 637 | 11.4 | 1.23 [1.01, 1.49] | 0.035 |  | 1.25 [1.03, 1.51] | 0.026 |
| Q4 | 658 | 11.9 | 1.34 [1.04, 1.74] | 0.024 |  | 1.37 [1.06, 1.78] | 0.016 |
|  |  |  |  |  |  |  |  |
| SD |  |  |  |  |  |  |  |
| Q1 | 538 | 9.6 | REF. |  |  | REF. |  |
| Q2 | 632 | 11.5 | 1.14 [0.99, 1.31] | 0.062 |  | 1.16 [1.01, 1.34] | 0.038 |
| Q3 | 646 | 11.7 | 1.22 [1.01, 1.48] | 0.040 |  | 1.29 [1.06, 1.57] | 0.013 |
| Q4 | 663 | 12.2 | 1.31 [1.12, 1.69] | 0.038 |  | 1.45 [1.10, 1.90] | 0.008 |
| Abbreviations: cVIM: Corrected variability independent of mean. HR: Hazard ratio. CI: confidence interval.  Model 1: Adjusted for age, sex and race  Model 2: Adjusted for age, sex, race, allocation to glycemia treatment arm, duration of diabetes, mean LDL, mean HDL, mean triglyceride, baseline BMI, cigarette smoking, insulin, statin, fibrate and other lipid medications | | | | | | | |

**Supplementary Table 6** Quartiles of lipid variability (cVIM) measures and risk of neuropathy after excluding participants who developed serious adverse events within the first 18month of follow-up

| Variables & categories | Events | Incidence rate / 100 person-yr | HR [95% CI] | P-value |  | HR [95% CI] | P-value |
| --- | --- | --- | --- | --- | --- | --- | --- |
| HDL Cholesterol |  |  | Model 1 |  |  | Model 2 |  |
| Q1 | 568 | 10.8 | REF. |  |  | REF. |  |
| Q2 | 617 | 11.5 | 1.15 [1.00, 1.32] | 0.044 |  | 1.18 [1.02, 1.35] | 0.021 |
| Q3 | 633 | 11.8 | 1.26 [1.04, 1.52] | 0.019 |  | 1.28 [1.06, 1.55] | 0.011 |
| Q4 | 631 | 11.6 | 1.33 [1.03, 1.71] | 0.030 |  | 1.36 [1.05, 1.76] | 0.019 |
|  |  |  |  |  |  |  |  |
| LDL Cholesterol |  |  | Model 1 |  |  | Model 2 |  |
| Q1 | 586 | 11.1 | REF. |  |  | REF. |  |
| Q2 | 623 | 11.8 | 1.13 [0.99, 1.30] | 0.078 |  | 1.12 [0.97, 1.28] | 0.116 |
| Q3 | 628 | 11.6 | 1.19 [0.99, 1.44] | 0.069 |  | 1.16 [0.96, 1.41] | 0.124 |
| Q4 | 612 | 11.1 | 1.22 [0.94, 1.57] | 0.132 |  | 1.16 [0.90, 1.51] | 0.253 |
|  |  |  |  |  |  |  |  |
| Triglyceride |  |  | Model 1 |  |  | Model 2 |  |
| Q1 | 555 | 10.3 | REF. |  |  | REF. |  |
| Q2 | 642 | 12.3 | 1.32 [1.15, 1.52] | <0.001 |  | 1.32 [1.15, 1.52] | <0.001 |
| Q3 | 635 | 11.7 | 1.34 [1.11, 1.63] | 0.003 |  | 1.35 [1.11, 1.64] | 0.002 |
| Q4 | 617 | 11.4 | 1.44 [1.11, 1.87] | 0.006 |  | 1.45 [1.12, 1.89] | 0.005 |
|  |  |  |  |  |  |  |  |
| Remnant Cholesterol |  |  | Model 1 |  |  | Model 2 |  |
| Q1 | 548 | 10.2 | REF. |  |  | REF. |  |
| Q2 | 657 | 12.7 | 1.30 [1.14, 1.50] | <0.001 |  | 1.30 [1.13, 1.49] | <0.001 |
| Q3 | 614 | 11.3 | 1.20 [0.99, 1.46] | 0.064 |  | 1.21 [1.00, 1.47] | 0.052 |
| Q4 | 630 | 11.6 | 1.31 [1.01, 1.70] | 0.041 |  | 1.33 [1.03, 1.73] | 0.032 |
| Abbreviations: cVIM: Corrected variability independent of mean. HR: Hazard ratio. CI: confidence interval.  Model 1: Adjusted for age, sex and race  Model 2: Adjusted for age, sex, race, allocation to glycemia treatment arm, duration of diabetes, mean LDL, mean HDL, mean triglyceride, baseline BMI, cigarette smoking, insulin, statin, fibrate and other lipid medications | | | | | | | |

**Supplementary Table 7** Quartiles of lipid variability measures and risk of retinopathy

| Variables & categories | Events | Incidence rate / 100 person-yr | HR [95% CI] | P-value |  | HR [95% CI] | P-value |
| --- | --- | --- | --- | --- | --- | --- | --- |
| HDL Cholesterol |  |  | Model 1 |  |  | Model 2 |  |
| **CV** |  |  |  |  |  |  |  |
| Q1 | 675 | 9.2 | REF. |  |  | REF. |  |
| Q2 | 691 | 9.1 | 1.06 [0.92, 1.21] | 0.432 |  | 1.08 [0.94, 1.24] | 0.267 |
| Q3 | 714 | 9.4 | 1.10 [0.92, 1.33] | 0.290 |  | 1.12 [0.93, 1.36] | 0.219 |
| Q4 | 733 | 9.7 | 1.22 [0.95, 1.55] | 0.118 |  | 1.23 [0.96, 1.58] | 0.097 |
|  |  |  |  |  |  |  |  |
| **SD** |  |  |  |  |  |  |  |
| Q1 | 645 | 8.7 | REF. |  |  | REF. |  |
| Q2 | 687 | 9.1 | 1.10 [0.96, 1.26] | 0.173 |  | 1.12 [0.98, 1.29] | 0.105 |
| Q3 | 752 | 10.0 | 1.16 [0.97, 1.40] | 0.109 |  | 1.18 [0.97, 1.42] | 0.091 |
| Q4 | 729 | 9.6 | 1.12 [0.87, 1.44] | 0.361 |  | 1.12 [0.87, 1.45] | 0.370 |
|  |  |  |  |  |  |  |  |
| LDL Cholesterol |  |  | Model 1 |  |  | Model 2 |  |
| **CV** |  |  |  |  |  |  |  |
| Q1 | 678 | 9.3 | REF. |  |  | REF. |  |
| Q2 | 710 | 9.6 | 1.01 [0.88, 1.15] | 0.906 |  | 1.02 [0.89, 1.17] | 0.726 |
| Q3 | 706 | 9.2 | 1.01 [0.84, 1.22] | 0.877 |  | 1.04 [0.86, 1.25] | 0.683 |
| Q4 | 719 | 9.3 | 0.98 [0.76, 1.25] | 0.864 |  | 0.99 [0.77, 1.27] | 0.948 |
|  |  |  |  |  |  |  |  |
| **SD** |  |  |  |  |  |  |  |
| Q1 | 684 | 9.4 | REF. |  |  | REF. |  |
| Q2 | 700 | 9.4 | 1.02 [0.89, 1.16] | 0.820 |  | 1.02 [0.89, 1.17] | 0.753 |
| Q3 | 706 | 9.2 | 1.01 [0.84, 1.84] | 0.900 |  | 1.01 [0.84, 1.22] | 0.879 |
| Q4 | 723 | 9.4 | 1.02 [0.80, 1.31] | 0.873 |  | 1.02 [0.79, 1.32] | 0.869 |
|  |  |  |  |  |  |  |  |
| Triglyceride |  |  | Model 1 |  |  | Model 2 |  |
| **CV** |  |  |  |  |  |  |  |
| Q1 | 664 | 9.0 | REF. |  |  | REF. |  |
| Q2 | 714 | 9.5 | 1.09 [0.95, 1.25] | 0.223 |  | 1.09 [0.95, 1.25] | 0.211 |
| Q3 | 735 | 9.6 | 1.17 [0.97, 1.41] | 0.095 |  | 1.17 [0.97, 1.41] | 0.110 |
| Q4 | 700 | 9.3 | 1.21 [0.94, 1.55] | 0.132 |  | 1.26 [0.98, 1.62] | 0.073 |
|  |  |  |  |  |  |  |  |
| **SD** |  |  |  |  |  |  |  |
| Q1 | 698 | 9.5 | REF. |  |  | REF. |  |
| Q2 | 707 | 9.4 | 0.98 [0.86, 1.12] | 0.799 |  | 1.01 [0.89, 1.16] | 0.831 |
| Q3 | 728 | 9.6 | 1.02 [0.85, 1.23] | 0.802 |  | 1.09 [0.90, 1.32] | 0.353 |
| Q4 | 680 | 8.9 | 1.00 [0.78, 1.29] | 0.980 |  | 1.13 [0.87, 1.49] | 0.360 |
|  |  |  |  |  |  |  |  |
| Remnant Cholesterol |  |  | Model 1 |  |  | Model 2 |  |
| **CV** |  |  |  |  |  |  |  |
| Q1 | 653 | 8.9 | REF. |  |  | REF. |  |
| Q2 | 711 | 9.5 | 1.16 [1.01, 1.32] | 0.037 |  | 1.16 [1.01, 1.33] | 0.037 |
| Q3 | 732 | 9.6 | 1.19 [0.98, 1.43] | 0.074 |  | 1.17 [0.97, 1.42] | 0.093 |
| Q4 | 717 | 9.5 | 1.23 [0.96, 1.58] | 0.101 |  | 1.26 [0.98, 1.62] | 0.071 |
|  |  |  |  |  |  |  |  |
| **SD** |  |  |  |  |  |  |  |
| Q1 | 698 | 9.5 | REF. |  |  | REF. |  |
| Q2 | 703 | 9.4 | 0.98 [0.85, 1.12] | 0.729 |  | 1.00 [0.88, 1.15] | 0.948 |
| Q3 | 725 | 9.5 | 1.06 [0.88, 1.27] | 0.549 |  | 1.14 [0.94, 1.38] | 0.186 |
| Q4 | 687 | 9.0 | 1.05 [0.82, 1.34] | 0.708 |  | 1.17 [0.90, 1.52] | 0.242 |
| Abbreviations: cVIM: Corrected variability independent of mean. HR: Hazard ratio. CI: confidence interval.  Model 1: Adjusted age, sex and race  Model 2: Adjusted for age, sex, race, allocation to glycemia treatment arm, BP vs lipid treatment arm, duration of diabetes, mean LDL, mean HDL, mean triglyceride, mean systolic BP, baseline BMI, insulin, statin, fibrate and other lipid medications | | | | | | | |

**Supplementary Table 8** Quartiles of lipid variability (cVIM) measures and risk of retinopathy after excluding participants who developed serious adverse events within the first 18month of follow-up

| Variables & categories | Events | Incidence rate / 100 person-yr | HR [95% CI] | P-value |  | HR [95% CI] | P-value |
| --- | --- | --- | --- | --- | --- | --- | --- |
| HDL Cholesterol |  |  | Model 1 |  |  | Model 2 |  |
| Q1 | 583 | 8.9 | REF. |  |  | REF. |  |
| Q2 | 616 | 9.3 | 1.09 [0.96, 1.25] | 0.195 |  | 1.12 [0.97, 1.28] | 0.117 |
| Q3 | 616 | 9.1 | 1.12 [0.93, 1.35] | 0.232 |  | 1.14 [0.95, 1.38] | 0.163 |
| Q4 | 633 | 9.6 | 1.24 [0.97, 1.59] | 0.090 |  | 1.25 [0.97, 1.61] | 0.079 |
|  |  |  |  |  |  |  |  |
| LDL Cholesterol |  |  | Model 1 |  |  | Model 2 |  |
| Q1 | 598 | 9.2 | REF. |  |  | REF. |  |
| Q2 | 616 | 9.4 | 1.05 [0.91, 1.20] | 0.517 |  | 1.06 [0.92, 1.21] | 0.418 |
| Q3 | 613 | 9.2 | 1.01 [0.84, 1.22] | 0.896 |  | 1.03 [0.86, 1.24] | 0.749 |
| Q4 | 621 | 9.2 | 1.02 [0.80, 1.30] | 0.884 |  | 1.03 [0.81, 1.33] | 0.795 |
|  |  |  |  |  |  |  |  |
| Triglyceride |  |  | Model 1 |  |  | Model 2 |  |
| Q1 | 575 | 8.8 | REF. |  |  | REF. |  |
| Q2 | 607 | 9.1 | 1.17 [0.94, 1.23] | 0.299 |  | 1.07 [0.93, 1.23] | 0.348 |
| Q3 | 645 | 9.7 | 1.19 [0.99, 1.43] | 0.069 |  | 1.17 [0.97, 1.41] | 0.103 |
| Q4 | 621 | 9.3 | 1.19 [0.93, 1.53] | 0.169 |  | 1.20 [0.93, 1.54] | 0.164 |
|  |  |  |  |  |  |  |  |
| Remnant Cholesterol |  |  | Model 1 |  |  | Model 2 |  |
| Q1 | 572 | 8.7 | REF. |  |  | REF. |  |
| Q2 | 596 | 9.0 | 1.07 [0.94, 1.23] | 0.317 |  | 1.08 [0.94, 1.24] | 0.282 |
| Q3 | 658 | 9.9 | 1.23 [1.02, 1.48] | 0.029 |  | 1.21 [1.03, 1.46] | 0.046 |
| Q4 | 622 | 9.3 | 1.22 [0.95, 1.56] | 0.125 |  | 1.23 [0.96, 1.58] | 0.106 |
| Abbreviations: cVIM: Corrected variability independent of mean. HR: Hazard ratio. CI: confidence interval.  Model 1: Adjusted for age, sex and race  Model 2: Adjusted for age, sex, race, allocation to glycemia treatment arm, BP vs lipid treatment arm, duration of diabetes, mean LDL, mean HDL, mean triglyceride, mean systolic BP, baseline BMI, insulin, statin, fibrate and other lipid medications | | | | | | | |

Supplementary Table 9. Lipid variability and risk of microvascular complications adjusting for HbA1c and BP variability

| Variables & categories | Nephropathy |  | Neuropathy |  |  | Retinopathy |  |
| --- | --- | --- | --- | --- | --- | --- | --- |
|  | HR [95% CI] | *P-*value | HR [95% CI] | *P-*value |  | HR [95% CI] | *P*-value |
| HDL Cholesterol |  |  |  |  |  |  |  |
| Q1 | REF. |  | REF. |  |  | REF. |  |
| Q2 | 1.18 [1.03, 1.36] | 0.020 | 1.18 [1.03, 1.35] | 0.020 |  | 1.10 [0.96, 1.26] | 0.183 |
| Q3 | 1.21 [1.00, 1.46] | 0.055 | 1.24 [1.02, 1.50] | 0.029 |  | 1.11 [0.92, 1.34] | 0.280 |
| Q4 | 1.45 [1.12, 1.86] | 0.004 | 1.33 [1.03, 1.72] | 0.028 |  | 1.19 [0.93, 1.53] | 0.175 |
|  |  |  |  |  |  |  |  |
| LDL Cholesterol |  |  |  |  |  |  |  |
| Q1 | REF. |  | REF. |  |  | REF. |  |
| Q2 | 1.10 [0.96, 1.27] | 0.163 | 1.12 [0.97, 1.28] | 0.114 |  | 1.04 [0.91, 1.20] | 0.526 |
| Q3 | 1.15 [0.95, 1.39] | 0.152 | 1.16 [0.96, 1.41] | 0.124 |  | 1.01 [0.84, 1.22] | 0.916 |
| Q4 | 1.13 [0.88, 1.46] | 0.346 | 1.13 [0.87, 1.46] | 0.366 |  | 0.99 [0.77, 1.28] | 0.962 |
|  |  |  |  |  |  |  |  |
| Triglyceride |  |  |  |  |  |  |  |
| Q1 | REF. |  | REF. |  |  | REF. |  |
| Q2 | 1.13 [0.98, 1.30] | 0.090 | 1.33 [1.16, 1.53] | <0.001 |  | 1.06 [0.93, 1.22] | 0.384 |
| Q3 | 1.33 [1.10, 1.61] | 0.003 | 1.34 [1.10, 1.63] | 0.003 |  | 1.15 [0.95, 1.38] | 0.148 |
| Q4 | 1.45 [1.12, 1.86] | 0.004 | 1.46 [1.13, 1.90] | 0.004 |  | 1.16 [0.90, 1.49] | 0.254 |
|  |  |  |  |  |  |  |  |
| Remnant Cholesterol |  |  |  |  |  |  |  |
| Q1 | REF. |  | REF. |  |  | REF. |  |
| Q2 | 1.08 [0.94, 1.24] | 0.270 | 1.31 [1.14, 1.50] | <0.001 |  | 1.07 [0.93, 1.22] | 0.368 |
| Q3 | 1.32 [1.09, 1.59] | 0.004 | 1.21 [1.00, 1.47] | 0.050 |  | 1.18 [0.98, 1.42] | 0.083 |
| Q4 | 1.34 [1.04, 1.73] | 0.022 | 1.33 [1.03, 1.73] | 0.029 |  | 1.19 [0.92, 1.53] | 0.182 |
| Abbreviations: cVIM: Corrected variability independent of mean. HR: Hazard ratio. CI: confidence interval.  Model for nephropathy was adjusted for age, sex, race, glycemia treatment arm, BP vs lipid treatment arm, duration of diabetes, mean LDL, mean HDL, mean triglyceride, baseline eGFR, baseline BMI, cardiovascular disease history, antihypertensive use, insulin, statin, fibrate, other lipid medication, cVIM HbA1c, and cVIM SBP  Model for neuropathy was adjusted for age, sex, race, allocation to glycemia treatment arm, BP vs lipid treatment arm, duration of diabetes, mean LDL, mean HDL, mean triglyceride, baseline BMI, insulin, statin, fibrate, other lipid medications, cVIM HbA1c, and cVIM SBP  Model for retinopathy was adjusted for age, sex, race, allocation to glycemia treatment arm, BP vs lipid treatment arm, duration of diabetes, mean LDL, mean HDL, mean triglyceride, baseline BMI, insulin, statin, fibrate, other lipid medications, cVIM HbA1c, and cVIM SBP | | | | | | | |
